# Orange emissive carbon dots for colorimetric and fluorescent sensing of 2,4,6-trinitrophenol by fluorescence conversion†

**DOI:** 10.1039/c8ra01678j

**Published:** 2018-04-30

**Authors:** Guojuan Ren, Liying Yu, Baoya Zhu, Mingyu Tang, Fang Chai, Chungang Wang, Zhongmin Su

**Affiliations:** Key Laboratory of Photochemical Biomaterials and Energy Storage Materials, Colleges of Heilongjiang Province, College of Chemistry and Chemical Engineering, Harbin Normal University Harbin 150025 P. R. China fangchai@gmail.com; Faculty of Chemistry, Northeast Normal University Changchun 130024 P. R. China wangcg925@nenu.edu.cn zmsu@nenu.edu.cn

## Abstract

In this study, infrequent orange carbon nanodots (CNDs) were applied as a dual-readout probe for the effective colorimetric and fluorescent detection of 2,4,6-trinitrophenol (TNP). The orange fluorescence could be rapidly and selectively quenched by TNP, and the colorimetric response from the original pink color to blue could also be captured immediately by the naked eye. A limit of detection of 0.127 μM for TNP was estimated by the fluorescent method and 5 × 10^−5^ M by visualized detection. Interestingly, the fluorescence of the CNDs with TNP gradually transitioned from orange to green upon irradiation by a UV lamp, and the colorimetric response transitioned from pink to blue to colorless, which ensured effective multi-response detection of TNP. In addition, the CNDs exhibited bright fluorescence, excellent biocompatibility and low toxicity, making them high-quality fluorescent probes for cellular imaging.

## Abstract 2

We have described a colorimetric and fluorescent dual-readout probe with a strong and sensitive response towards TNP.

## Graphical abstract

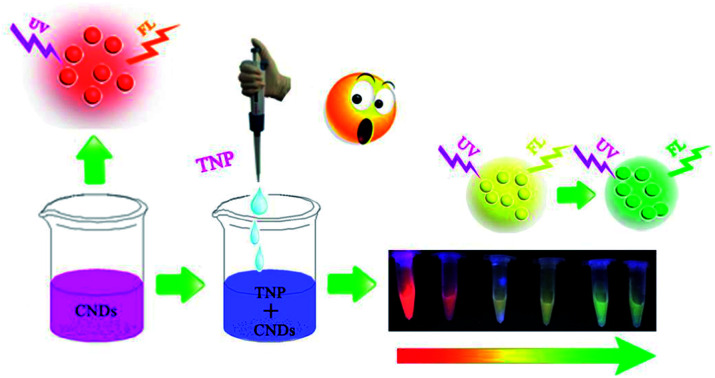

## 1. Introduction

As a member of the nano-carbon family, CNDs have received great attention in recent years due to their ease of preparation.^1^ Within this large family, CNDs can be synthesized by relatively simple procedures and it is easy to perform surface modifications; additionally, CNDs have high quantum yield, good aqueous dispersibility and low toxicity compared with traditional fluorescent dyes.^2,3^ Particularly, as a consequence of tunable fluorescence and good biocompatibility, CNDs have become an attractive alternative for novel fluorescence markers, fluorescent probes, and optical sensors.^4^ Fluorescent probes exhibit various potential applications such as the sensitive detection of metal ions,^5,6^ pesticides,^7^ explosives^8,9^ and biological molecules.^10,11^ Among them, as explosives, which are inflammable dangerous goods, are security concerns worldwide, their detection is of great importance. Nitroaromatic compounds are highly dangerous and environmentally harmful substances, which should be paid attention to by the society. 2,4,6-Trinitrophenol (TNP), also called picric acid, is a type of explosive with low safety coefficient and strong explosive capacity.^12^ In addition, serious environmental pollution caused by the use TNP in the dye industry has contaminated the soil and water; inhaling, ingesting or touching TNP can cause skin irritation, weakness, headache, aplastic anemia and liver injury.^13^ Moreover, distinguishing between the various nitroaromatic explosives is difficult due to their similarity, which has limited the practical application of many probes. Therefore, it is urgently required to develop a simple, rapid, highly selective and sensitive detection method for TNP.

Recently, a variety of excellent sensors for analyzing explosives, particularly TNP, were reported.^14,15^ For example, Kansal *et al.* achieved selective detection of TNP using N-doped graphene quantum dots, with a detection limit of 420 nM.^16^ Huang *et al.* developed terbium-doped blue carbon dots as a fluorescence probe for TNP detection; TNP can be detected with limit of 200 nM level in aqueous solution.^17^ Yan *et al.* developed P-doped carbon dots, which act as a nanosensor for trace TNP detection, with a detection limit of 16.9 nM.^18^ To date, many successful methods have been developed that utilize several techniques, including mass spectrometry,^19^ X-ray imaging,^20^ and electrochemical methods;^21^ however, they can be time-consuming and complicated, and generally require a complex labelling process. Compared with these methods, fluorescence-based detection exhibits several advantages such as rapid response times, high sensitivity, real-time monitoring and specificity.^22^ Similarly, the colorimetric method has also received much attention due to their direct visible detection without the need for complicated instruments. Therefore, based on the advantages of colorimetric and fluorescent methods, the dual-function detection method, which combines these two methods with high sensitivity, multiple live visualized results and low environmental interference impact, has attracted a lot of attention.^23,24^ It is practical to explore a novel dual functional fluorescent and colorimetric probe for visual determination of TNP.

**Scheme 1:**
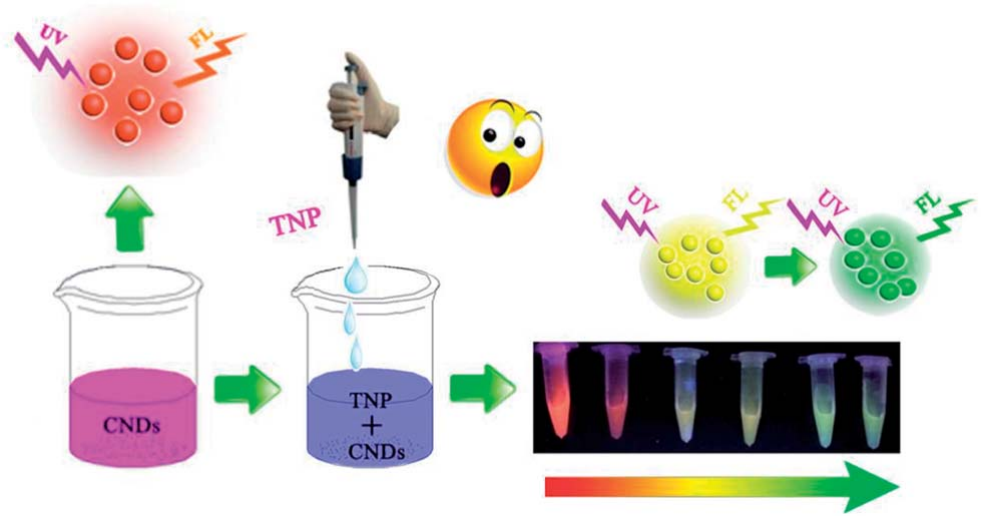
Schematic diagram of the detection of TNP by CNDs.

Inspired by this, we synthesized CNDs according to the reported literature. The CNDs exhibited bright orange emission under an excitation wavelength of 540 nm; the orange fluorescent CNDs were explored in both bioimaging- and biosensor-based applications. Since carbon composes the backbone of biomolecules it is not surprising that CNDs are more biocompatible than other materials.^25^ Many researchers have reported high efficiency of CNDs in cells imaging due to their favorable biocompatibility.^26–28^ However, most of them exhibited blue or green emission. In this study, the orange emissive CNDs were prepared and applied as a fluorescent probe. We designed it to conjugate with folic acid (FA) and used it in live-cell fluorescent imaging. In this study, significant efforts have been made to develop a CNDs-based fluorescent and colorimetric dual-readout sensor for TNP detection with high sensitivity and selectivity (Scheme 1).

## 2. Experimental

### 2.1 Chemicals and characterization

The reagents used in the experiments were of analytical grade and used without further purification. Citric acid, urea, 2-nitrophenol (2-NP) (AR, 98%), 3-nitrophenol (3-NP) (AR, 98%), 4-nitrophenol (4-NP) (98%), and 2,4,6-trinitrophenol (TNP) (AR, 98%) were purchased from Aladdin (Shanghai). Dimethylformamide (DMF) was purchased from Fenghua Chemical Reagent Company (Tianjin). 3-Nitrotoluene (3-NT) (98%), nitrobenzene (NB) (AR, 98%) and toluene (98%) were purchased from the Beijing Chemical Reagent Company (Beijing, China). 2,4-Dinitrotoluene (DNT) (98%) and 2,4,6-trinitrotoluene (TNT) (≥98%) were supplied by the National Security Department of China. Milli-Q water was used to prepare all the solutions in this study.

Transmission electron microscopy (TEM) was used to record the size and morphology of the CNDs with a JEOLFETEM-2100 operating at an accelerating voltage of 200 kV. Fourier transform infrared spectra (FTIR) were recorded with a JASCOFT/IR-420. Fluorescence spectra were conducted with a PerkinElmer LS-55 fluorescence spectrometer. UV-vis absorption spectra were performed using a U-2550 spectrophotometer (SHIMADZU, Japan) at room temperature. X-ray photoelectron spectra (XPS) were determined with an AXIS Ultra DLD spectrometer.

### 2.2 CNDs synthesis

CNDs were synthesized according to the reported literature.^29^ In this experiment, citric acid (1 g) and urea (2 g) were heated under solvothermal conditions in 10 mL DMF at 160 °C for 6 h. The brown solution was cooled to room temperature and mixed with 20 mL 50 mg mL^−1^ NaOH solution for 1 min and then centrifuged at 12 000 rpm for 10 min. The dark precipitate was dissolved in water and centrifuged twice to remove alkali and residual salts, and then dried by freeze drying.

### 2.3 CNDs as probes for 2,4,6-trinitrophenol sensing

In brief, 0.001 g powder of CNDs was diluted with ultrapure water to a concentration of 36 μg mL^−1^ and was used as a colorimetric and fluorescent probe in the detection of TNP. Selectivity was achieved by adding ethanol solutions of other related analogues including toluene, 3-nitrotoluene (3-NT), nitrobenzene (NB), 2,4-dinitrotoluene (DNT), 4-nitrophenol (4-NP), 2,4,6-trinitrotoluene (TNT), 3-nitrophenol (3-NP), and 2-nitrophenol (2-NP) as alternatives to 2,4,6-trinitrophenol (TNP) with the CNDs probe with a volume ratio of 1 : 1. After incubating for 1 min, the UV-vis spectra and fluorescence spectra of the samples were recorded. The blank was prepared by mixing 150 μL ethanol and 150 μL of the CNDs probe. The sensitivity was evaluated by investigating the colorimetric and fluorescent intensity of the CNDs with different concentrations (10^−3^–10^−9^ M) of TNP samples. In addition, experiments about the effects of interferents on the detection of TNP *via* UV irradiation were also investigated carefully.

### 2.4 MTT and cell imaging

The MTT assay and cell imaging were performed according to the reported experiments and methods detailed in the ESI.†

## 3. Results and discussion

### 3.1 Characteristics of CNDs

In this paper, we report a CND-based colorimetric and fluorescent dual functional probe with the activity of detection for TNP, as illustrated in Scheme 1. The as-prepared CNDs presented a vivid pink color and exhibited bright orange emission under UV light. When exposed to the explosive TNP, the color of the CNDs immediately changed from pink to blue; accordingly, the brightness of the CNDs disappeared. Interestingly, the quenched sample could be turned on with the help of UV irradiation, and the fluorescent color of the sample gradually transitioned from yellow to green with irradiation time; correspondingly, a colorimetric evolution occurred and the color of the sample turned from pink to blue and then to colorless. Inspired by this colorful diversification caused by TNP, the selection of CNDs as a dual readout visible sensor to quantify the existence of TNP was considered as a brilliant idea.

**Fig. 1:**
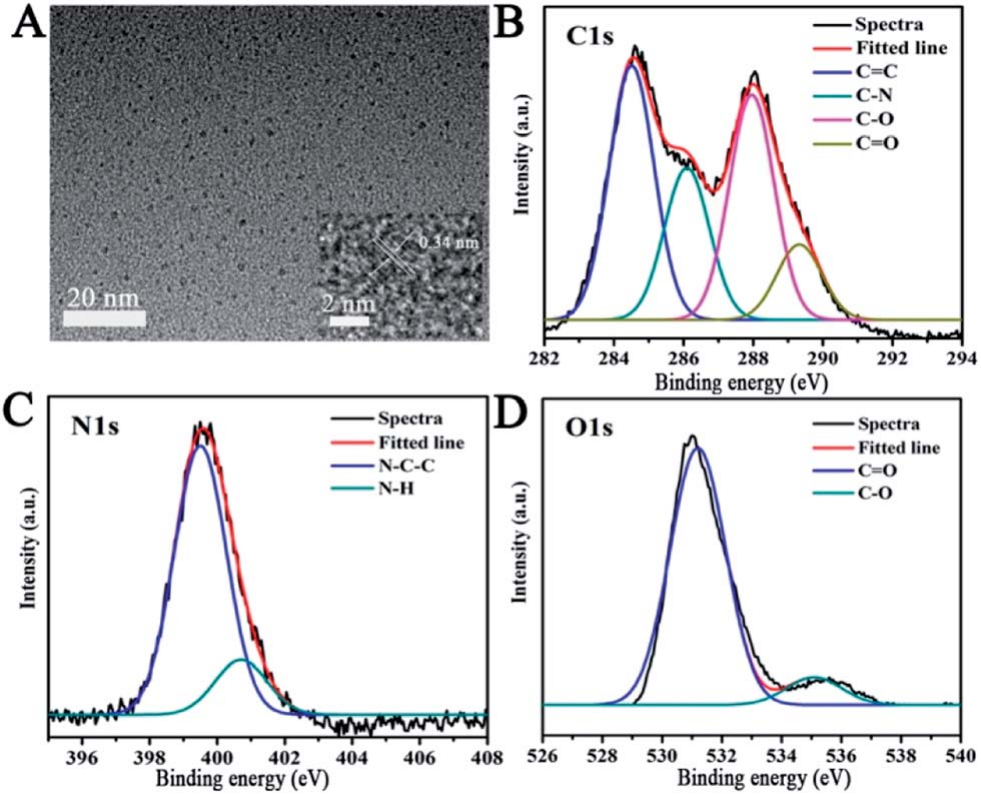
(A) TEM images of CNDs. Inset: high-resolution TEM image. The high-resolution XPS spectra of C1s (B), N1s (C) and O1s (D).

Systematic characterizations involving TEM, XPS, FTIR and FL decay curves were performed to investigate the physical/chemical properties of the CNDs. As presented in the TEM image, the CNDs have spherical shape with good monodispersion. The CNDs had a size distribution in the range of 1.74–3.48 nm with a mean value of around 1.87 nm (Fig. S1†). Well-resolved lattice fringes with an interplanar distance of 0.34 nm for CND are attributed to the (002) facet of graphitic carbon (inset of Fig. 1A), demonstrating excellent crystallinity of the CNDs.

The survey XPS spectrum was recorded to explore the chemical composition and chemical bonding of the CNDs. The peaks at binding energies of 284.6, 400.1 and 531.4 eV are shown in the XPS spectrum of the CNDs (Fig. S2†), indicating the presence of C, N and O in the product, respectively. The C1s XPS spectrum of the CNDs (Fig. 1B) showed four peaks at 284.3, 286.1, 287.9 and 289.2 eV, which can be attributed to C=C, C–N, C–O and C=O bonding, respectively. Additionally, the two peaks observed at 399.2 and 400.9 eV (Fig. 1C), which were associated with N–C–C, and N–H groups, indicate the existence of amide groups. The C=O signal present at 531.2 eV, and 535.1 eV can be attributed to the C–O bond (Fig. 1D) in the O1s XPS spectrum of the CNDs.^30–32^ The XPS attributions were further verified by the FTIR spectrum. The FTIR spectrum was obtained to determine the surface status information of the CNDs (Fig. S3†). The FTIR spectrum of the CNDs showed the absorption band at 3433 cm^−1^, which was assigned to *ν*(N–H). Two absorption peaks at 1631 cm^−1^ and 1394 cm^−1^, which were assigned to *ν*(C=C) and *ν*(C–H) stretching vibration, respectively, confirmed the successful synthesis of CNDs.

**Fig. 2:**
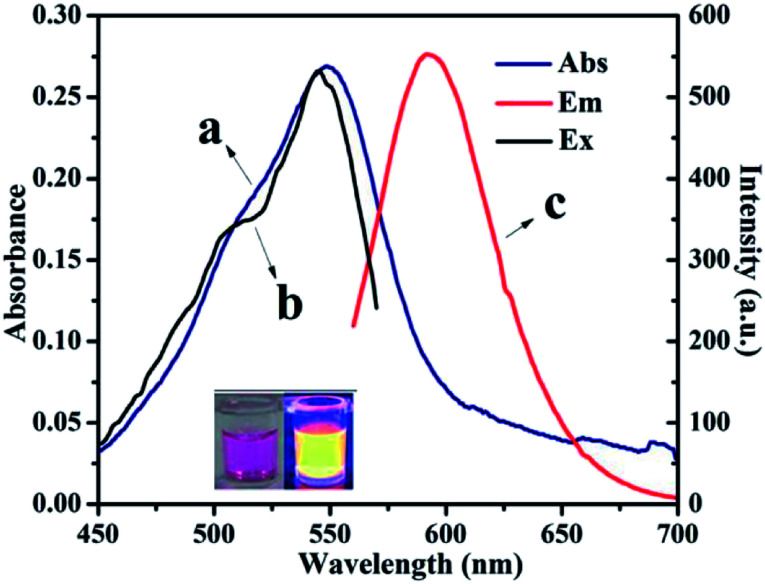
UV-vis spectra (a) and fluorescence spectra of CNDs dilute ethanol solution (*λ*_ex_: (b), *λ*_em_: (c)).

From fundamental and application viewpoints, fluorescence is one of the most fascinating features of CNDs. The fluorescence spectra of CNDs revealed that excitation (curve b) and emission (curve c) maxima were located at 540 and 590 nm, respectively, as shown in Fig. 2. The UV-vis absorption peak (curve a) was similar to the excitation peak, which corresponds to the pink color of the CNDs in daylight. The inset in Fig. 2 shows the optical photo of the CNDs upon irradiation with a 365 nm UV lamp, which clearly exhibited an orange fluorescent brightness.^33^ As shown in Fig. S4,† the FL decay curve was fitted with multi-exponential models. The fluorescence lifetime was 2.65 ns for the CNDs alone. The quantum yield was measured and calculated to be 43%, which was lower than the original reported literature.

### 3.2 Selectivity of CNDs

**Fig. 3:**
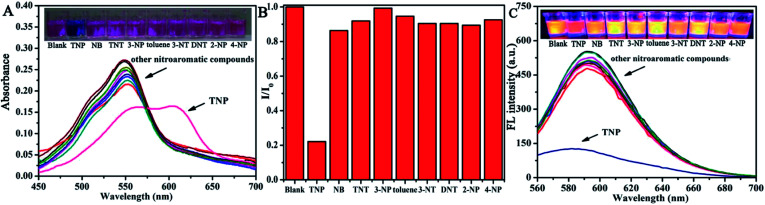
Selectivity towards various nitroaromatic compounds of the CNDs. (A) UV-vis absorption spectra of CNDs in ethanol–water in the presence of various analytes (10^−3^ M). Inset: color change. (B) Relative fluorescence emission intensity of CNDs in the presence of various analytes. (C) Fluorescence spectra of the probe with various analytes. Inset: color change.

For ultrasensitive colorimetric and fluorescent detection of TNP, the as-prepared CNDs were diluted and used without any further modification. Selectivity was a very significant parameter to evaluate the performance of the sensor.^34^ To examine the sensing selectivity of CNDs toward TNP, the effects of different types of nitroaromatic compounds, namely, toluene, NB, 3-NT, 4-NP, TNT, 2-NP, 3-NP, DNT and TNP, on both UV-vis absorbance and fluorescence were investigated (Fig. S5†). Fig. 3A shows the absorbance of the CNDs after adding different nitroaromatic compounds. When exposed to TNP, the absorbance at 548 nm of the CNDs significantly decreased and shifted from 548 nm to 605 nm. In addition, another absorbance peak emerged at 605 nm, which could be attributed to the formation of a compound; correspondingly, the color of the CNDs changed from original pink to blue (Fig. 3A inset).^35^ In contrast, other nitroaromatic compounds did not cause any distinct changes in both the absorbance and color of the CNDs (Fig. 3A and inset). The color change of the CNDs induced by TNP could be developed into a simple and fast colorimetric method for the detection of TNP with the naked eye.

Moreover, the CNDs were further used as a fluorescent probe to detect TNP; the fluorescent intensity changes of the CNDs in the presence of all nitroaromatic compounds were also tested under the same conditions. With the same concentration of all nitroaromatic compounds, the fluorescence intensity of CNDs was effectively quenched by TNP, while the others showed almost no interference with the fluorescence of CNDs. The ratio of the fluorescence emission of the sample *versus* the blank (*I*/*I*_0_) was calculated to intuitively illustrate the comparison (Fig. 3B). The ratio of other samples was at least 3-times higher than that of TNP, which indicates the high selectivity of the CNDs for TNP, which was manifested by the observation that the others samples did not interfere with TNP detection. Clearly, a distinguishable color change from the original orange could be observed upon the drastic decrease in the emission intensity at 590 nm, which was satisfactory for visual detection of TNP by the naked eye (Fig. 3C). The corresponding images along with the spectra after fluorescence quenching were also recorded and there is no significant difference observed compared to those of the blank. These results proved the high selectivity of the CND-based dual-readout colorimetric and fluorescent probe for the identification of TNP over other nitroaromatic compounds.

### 3.3 Sensitivity of CNDs

**Fig. 4:**
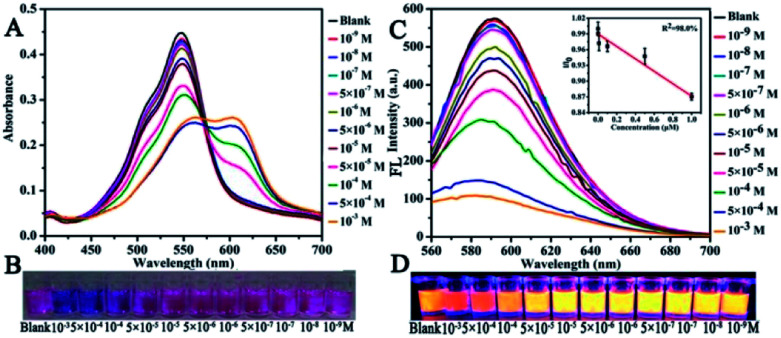
Detection of sensitivity: (A) UV-vis spectra and (C) fluorescence emission of CNDs in the presence of different concentrations of TNP (inset: linear curve of TNP). Photographs of the CNDs with different concentrations of TNP at (B) day light and (D) UV light.

The sensitivity of CNDs to TNP was tested by the colorimetric and fluorescent method, followed by calculation of the detection limit. The changes in the UV-vis spectra with concentration were monitored. The results of the detection of the volume ratio of the CNDs and TNP (1 : 1) with different concentrations are shown in Fig. 4A. The UV-vis spectra of these CNDs decreased with increasing concentrations of TNP. When the concentration of TNP was diluted to 5 × 10^−5^ M, a second absorption emerged, located at 605 nm, and the color changed from pink to purple accordingly. Upon increasing the concentration of TNP from 10^−9^ M to 10^−3^ M, the color of the CNDs dispersions gradually changed from pink to blue (from right to left, Fig. 4B), which was in accordance with the results from the spectra. Under the daylight condition, 5 × 10^−5^ M TNP in the solution could be easily detected by CNDs and observed by naked eyes.

The sensitivity of the CNDs probe towards TNP was also investigated by fluorescence spectroscopy. Serial samples of CNDs with various concentrations of TNP were analyzed, and fluorescence emission spectra were collected and compared at the same conditions. The fluorescence emission intensity of the CNDs decreased with the addition of TNP, as can be observed from Fig. 4C. The corresponding images of the CNDs with different concentrations of TNP under UV light (*λ*_ex_ = 365 nm) are provided in Fig. 4D, which are consistent with the fluorescence emission data. The brightness of the CNDs reduced with the addition of TNP, until the fluorescent emission was completely quenched. As shown in the inset in Fig. 4C, the plot of the relative fluorescence intensity (*I*/*I*_0_) against the concentration of TNP (0–1 μM) (where *I* and *I*_0_ are the FL intensities of the CNDs at 590 nm in the presence of TNP and blank, respectively) curves downward. The detection limit for TNP was determined to be 0.127 μM.^36^ Compared with some single fluorescent probe, the CNDs baseddual-readout probe exhibited much higher sensitivity in detection.^37–39^ Thus, the developed CNDs could create a simple and convenient platform for visualized quantitative detection of TNP activity with desirable sensitivity and high selectivity.

### 3.4 Effect of irradiation time

**Fig. 5:**
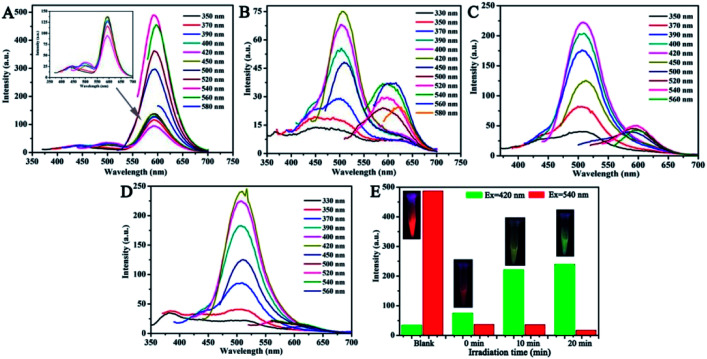
(A) Fluorescence spectra of CNDs at different excitation wavelengths. Inset: magnified areas. (B–D) Emission spectra of CNDs–TNP with different excitations under a UV lamp (365 nm) for different irradiation times (0 min, 10 min and 20 min). (E) Emission intensity histogram of four samples (A–D) excited at 420 nm and 540 nm, respectively. Inset: fluorescence color changes.

Interestingly, the fluorescence color of the CNDs gradually transitioned from orange to green after exposure to TNP with UV irradiation. Thus, the fluorescence emission of the CNDs was further investigated under UV radiation. The stable fluorescent emission of CNDs can be observed at 590 nm when excited at 350–580 nm (Fig. 5A). The emission centered at 590 nm was quenched by TNP; also, the weak emission of the CNDs shifted under other excited conditions (Fig. 5B). However, after exposure to UV irradiation (365 nm) for about 10 min, the emission of the sample turned on and the intensity of emission at 505 nm increased, which can also be seen by comparing the images (inset of Fig. 5E). The maximum emission was centered at 505 nm when excited at 350–450 nm (Fig. 5C). When the exposure time reached 20 min, the intensity of the emission continued to rise, and the emission peak was transferred to 505 nm at the highest excitation wavelength; the corresponding image exhibited bright green light (Fig. 5D). Above results make it clear when with the assistant of UV irradiation, the emission of the CNDs with TNP transferred from 590 nm to 505 nm, and the process of transform underwent the quenching and the energy transfer with the brightness turned from orange to dark yellow and green. The emission of the CNDs at 590 nm and 505 nm was observed after excitation at 540 nm and 420 nm, respectively, and the intensity of two emissions are expressed by the red and green columns, respectively, (Fig. 5E). As depicted in Fig. 5E, without TNP, the fluorescent intensity at 590 nm was over 20-fold stronger than at 505 nm, and the bright orange emission can be detected by the naked eyes (Fig. 5E inset). In the presence of TNP, the two emissions are completely quenched; correspondingly, the sample exhibited a dark-red emission (Fig. 5E inset). Upon irradiation for 10 min, the intensity of CNDs at 505 nm significantly increased; however, emission at 590 nm was still absent. The fluorescent color of the CNDs changed from orange to yellow-green (Fig. 5E inset). With a delay in irradiation, the fluorescence of the CNDs underwent further transformation. The intensity at 505 nm was sustained and increased, while the fluorescence at 590 nm decreased, which eventually led to a green fluorescent color. The above results indicate that TNP can cause a shift in the fluorescence color of CNDs from orange to green upon irradiation.

#### 3.4.1 Selectivity with irradiation

**Fig. 6:**
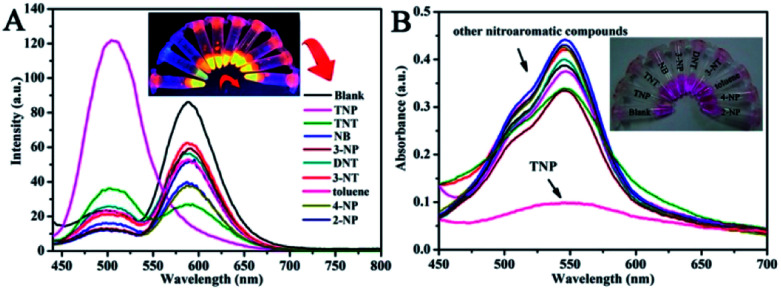
Selectivity of CNDs towards TNP over various other nitroaromatic compounds under a UV lamp for 20 min. (A) Fluorescence spectra of the probe with various analytes. Inset: color change. (B) UV-vis absorption spectra of the CNDs in ethanol–water in the presence of various analytes (10^−3^ M). Inset: color change.

In order to demonstrate the effect of TNP on the CNDs after an irradiation time of 20 min, we selected other explosives for comparison (*λ*_ex_ = 420 nm). Under the UV lamp irradiation, it was noticeable that TNP and TNT could cause a change in the fluorescence color and a decrease in the fluorescence intensity, respectively. The fluorescence color of the CNDs solution with TNP became green, and the fluorescence intensity of the CNDs with TNT decreased. To test this effect on selectivity, the fluorescence and UV-vis spectra of the mixture of CNDs with all explosives after irradiation for 20 min were recorded and displayed in Fig. 6. Clearly, only TNP changed the fluorescence and absorption of the CNDs compared with the others. In the fluorescence spectra, the emission of CND–TNP shifted from 590 nm to 505 nm, resulting in the orange emission turning green (Fig. 6A). Moreover, the intensity of absorption decreased, and the corresponding color turned from pink to blue and finally to colorless upon irradiation (Fig. 6B). It is worth noting that after irradiation, the fluorescence intensity of the TNT sample distinctly changed, which did not occur without irradiation. As shown in the fluorescence spectra, the emission intensity of the TNT sample distinctly changed compared with the blank. The transformation between the dual emission peaks occurred in the sample of TNT. The intensity of emission at 505 nm increased, while that at 590 nm reduced, which resulted in the fluorescent color transitioning from bright orange to orange-red. The irradiation induced change can also be observed from the darkening of pink color, as shown in Fig. 6B. Although, the effect of irradiation on the TNT sample was not stronger than the TNP sample, the variation was distinct. Thus, irradiation improved the energy transfer of CNDs with TNP and TNT, which can be attributed to the generation of new complexes.^35^ Thus, upon irradiation, the CNDs can be treated as a dual probe in the detection of both TNP and TNT. Additionally, the distinction of TNP and TNT in the detection can be easily discerned.

#### 3.4.2 Sensitivity with irradiation

**Fig. 7:**
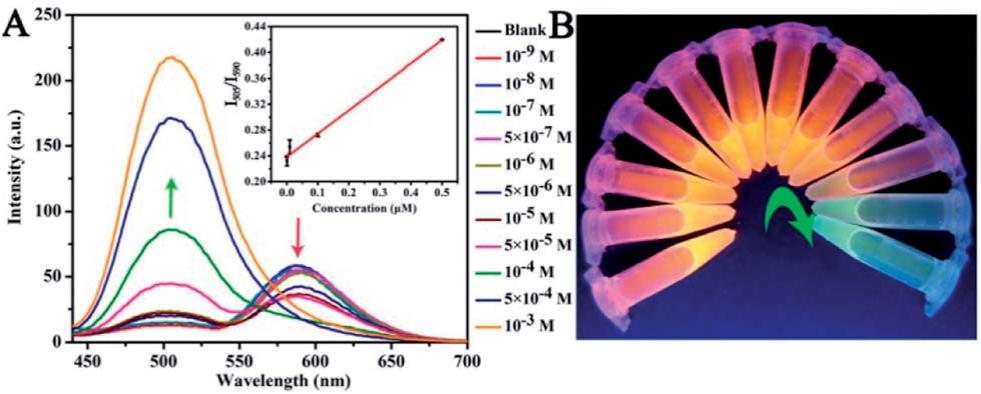
(A) The evolution of the fluorescence spectra of the mixture of CNDs with the addition of TNP. Inset: linear curve of TNP. (B) The photos showed the corresponding color evolutions under a UV lamp.

**Fig. 8:**
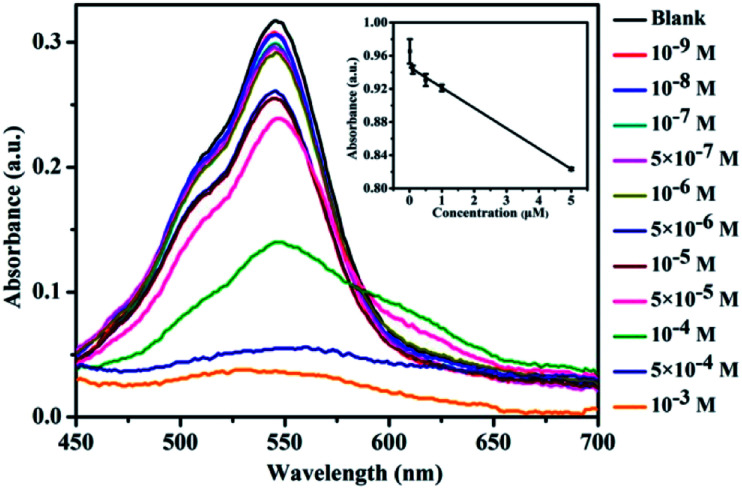
Absorption spectra of CNDs and various concentrations TNP mixture under UV lamp for 20 min. Inset: linear curve of TNP.

The detection of TNP and TNT after exposure to irradiation was evaluated systematically. With the addition of TNP to CNDs, we observed a concomitant decrease in intensity in orange emission at 590 nm, while the fluorescence intensity of the green emission from the CNDs–TNP at 505 nm increased (Fig. 7A). As shown in the inset, a linear relationship between the fluorescence intensity ratio (*I*_505_/*I*_590_) and TNP concentration in the range of 0–0.5 μM was observed. The detection limit for TNP was 44.4 nM (*R*^2^ = 0.99). In other words, the fluorescence color change of the CNDs could be used to determine the introduced TNP quantity at a fixed reaction time. Fig. 7B shows that the fluorescent colors of the CNDs gradually transformed from orange to green with the addition of TNP under the UV lamp for a few minutes. Moreover, with the increase in TNT concentration, the intensities of CNDs decreased accordingly, which allowed the visual detection of TNT. The limit of detection of 28.3 μM for TNT was achieved with a wide linear detection range of 5 to 1000 μM between the fluorescence intensity ratio (*I*_505_/*I*_590_) and TNT concentration (Fig. S6†). Though the structure of TNT is similar to that of TNP, there was no time-dependent fading of color. Thus, TNT and TNP could be distinguished quickly by the as-synthesized CNDs. Further, we incubated CNDs with different concentrations of TNP and exposed the samples to UV irradiation for about 20 min. The UV-vis absorption spectra of these CNDs samples suggested a dose-dependent decrease in the presence of TNP at 548 nm. There is a linear relationship from 0.01 to 5 μM between the relative absorption intensity (*A*_CND–TNP_/*A*_CND_) and the TNP concentration, with a detection limit of 0.523 μM (Fig. 8).

**Fig. 9:**
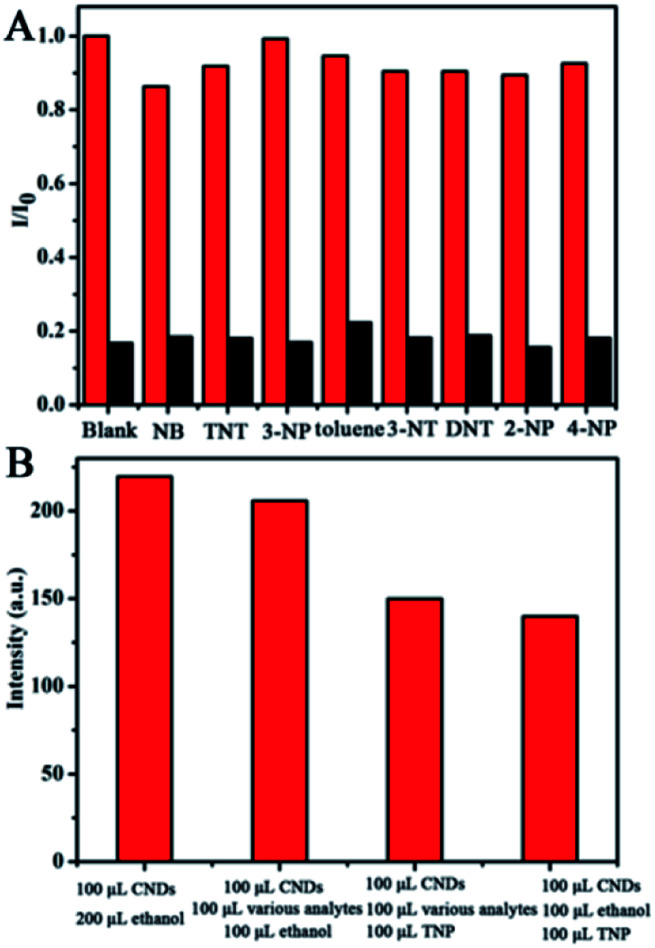
Effect of nitroaromatic explosives on the fluorescence of CNDs (the excitation wavelength is 540 nm). (A) The red bars represent the fluorescence emission of CNDs and nitroaromatic explosives, respectively. The black bars show the fluorescence that occurs upon addition of TNP to the solution containing CNDs and the corresponding nitroaromatic explosives, respectively. (B) Fluorescence response of CNDs to the mixture of all the nitroaromatic explosives without and with TNP.

To further test the specificity for TNP, the interference of other nitroaromatic explosives in TNP detection was investigated since nitroaromatic explosives are mixed in many cases. The other nitroaromatic explosives acted as interferents and the response of the CNDs to the mixed solution were tested under the same conditions. As depicted in Fig. 9A, the red columns represent the fluorescence emission of the CNDs when exposed to the nitroaromatics, indicating that the CNDs retained good emission. When TNP was introduced to the above samples, the inductive TNP immediately quenched the fluorescence emission of CNDs (black column in Fig. 9A), which indicated that the interference of other nitroaromatics in the detection of TNP can be neglected. To evaluate the mixed nitroaromatics effect on the detection of TNP, the mixed nitroaromatics sample was also added into the CNDs. As shown in Fig. 9B, the addition of the mixed nitroaromatics (30% of volume) quenched the emission by only about 12.5%. Under the same condition, TNP (30% of volume) can quench the emission by about 37.5%. Hence, the detection of TNP should not be affected by the mixture of nitroaromatics. The corresponding research under UV irradiation has also been estimated; as depicted in Fig. S7,† the emission of CND shifted from 590 nm to 505 nm and was greatly enhanced with TNP, resulting in the orange emission turning green.

### 3.5 Investigation of the possible quenching mechanism

In order to illustrate the selective response of CNDs to TNP and TNT, the possible mechanism was investigated. A resonance energy transfer-based sensing strategy was considered to be a possible mechanism for the selective identification due to the colorimetric response and the transformation of the absorbance. However, there is no spectral overlap between the absorption spectrum of TNP and the excitation or emission spectrum of the CNDs (Fig. S8†). Therefore, we deduced that energy transfer is not the dominant quenching pathway.^40^ Another mechanism for fluorescence quenching depends on electron-transfer from the electron-rich CNDs to electron deficient TNP/TNT, which was similar with that reported in previous studies.^16,41^ TNP and TNT are typical electron-deficient compounds due to the strong electron-withdrawing effect of the three nitro groups. The CNDs with their electron-rich amino groups could effectively bond to TNP/TNT *via* intermolecular interactions. The electron transfers from the amino groups to the nitro groups between the CNDs and the nitroaromatic compounds to form a complex and the charge transfer complexing interaction would lead to transformation of the fluorescence of CNDs.^35^ Moreover, hydrogen bonding is demonstrated as the mode of interaction, which possibly facilitates effective charge-transfer, which is probably the reason why TNP and TNT can be distinguished.^22^

### 3.6 Application of CNDs in living cells

**Fig. 10:**
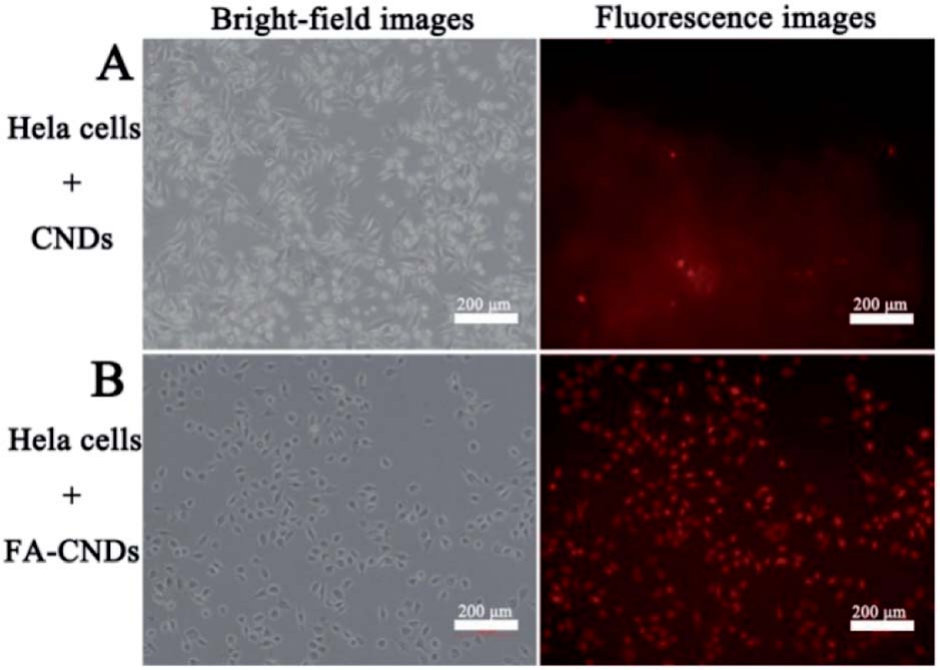
Bright-field (left) and (right) fluorescence images upon exposure of HeLa cells to green light after treatment with (A) the CNDs and (B) the FA–CNDs in cell medium for 3 h.

Considering the stable fluorescence and excellent biocompatibility of the CNDs, we investigated its significant potential in bioimaging applications. Herein, it was necessary to validate their benignity and cell compatibility *in vitro* before using CNDs for cell imaging. A methylthiazolyldiphenyltetrazolium bromide (MTT) assay was carried out to determine the cytotoxicity of the CNDs for HeLa cell imaging (Fig. S9†). There was limited influence of the CNDs on the cell viability of the HeLa cells after incubation for 24 h. The CNDs concentrations were significantly lower than those that could cause cell damage. This result suggests that CNDs can be used for bioimaging. In the cellular imaging experiments, the CNDs solution was incubated with HeLa cells at final concentration of 0.03125 mg mL^−1^ for 3 h.^42^Fig. 10 shows the fluorescence images of CNDs (with and without FA) pre-incubated with the HeLa cells for 3 h. The CNDs with FA (Fig. 10B) show more red emission than those without FA (Fig. 10A) in the intracellular region, which was ascribed to the fast uptake of these nanosized particles on FA modification. Recently, some studies have been carried out on grafting materials with FA for tracing the behaviour of folate receptor-mediated uptake by cancer cells due to its over-expression on many types of cancer cells.^43,44^ The FA-conjugated CNDs were then employed for targeted cell delivery. CNDs bioimaging of the cells were conducted *via* fluorescence microscopy. From the resulting images, it was found that the red emission was mainly located in the cytoplasmic regions, which demonstrated that CNDs could pass through cell membranes and give a detectable signal in a biological environment, thus exhibiting potential in bioimaging.

## 4. Conclusion

In summary, we reported a colorimetric and fluorescent dual-readout probe with strong and sensitive ON–OFF response toward TNP. On addition of TNP to CNDs, the fluorescence intensity of the CNDs could be immediately quenched, which could be detected colorimetrically by the naked eye with a detection limit for TNP as low as 5 × 10^−5^ M. The detection limit of 0.127 μM for CNDs was estimated by the fluorescence detection, which was much lower than the reported method. Furthermore, the differences in color change made it possible to detect TNT and TNP by the naked eyes on irradiating CNDs–TNP and CNDs–TNT with a UV lamp. In addition to their sensing applications, due to their stable optical properties and low cytotoxicity, the CNDs can also be used for cell labelling and tissue imaging. This study provided a successful paradigm to explore a simple and effective method to extend the applications of CNDs in bioimaging.

## Conflicts of interest

There are no conflicts of interest to declare.

## Supplementary Material

RA-008-C8RA01678J-s001
