# Supplementary material for: Orange emissive carbon dots for colorimetric and fluorescent sensing of 2,4,6-trinitrophenol by fluorescence conversion
Source: RSC Adv. 2018 Apr 30;8(29):16095–102. doi: 10.1039/c8ra01678j (PMC9080255; doi:10.1039/c8ra01678j)
Supplement: RA-008-C8RA01678J-s001 [file RA-008-C8RA01678J-s001.pdf]

Electronic Supplementary Material (ESI) for RSC Advances.
This journal is © The Royal Society of Chemistry 2018

Supporting Information

Orange emissive carbon dots for colorimetric and fluorescent sensing 2, 4, 6-trinitrophenol by fluorescence conversion

Guojuan Ren,[a] Liying Yu,[a] Baoya Zhu,[a] Mingyu Tang,[a] Fang Chai,*[a] Chungang Wang,*[b] Zhongmin Su*[b]

[a] Key Laboratory of Photochemical Biomaterials and Energy Storage Materials, Colleges of Heilongjiang Province, College of Chemistry and Chemical Engineering, Harbin Normal University, Harbin 150025, P. R. China

E-mail: fangchai@gmail.com

[b] Faculty of Chemistry, Northeast Normal University, Changchun 130024, P. R. China

E-mail: wangcg925@nenu.edu.cn; zmsu@nenu.edu.cn

* corresponding author

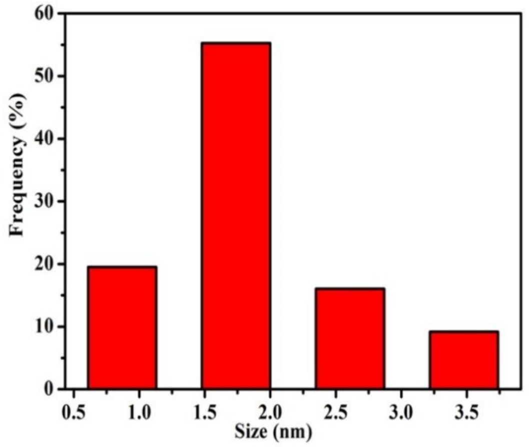

Fig. S1. Size distribution of CNDs.

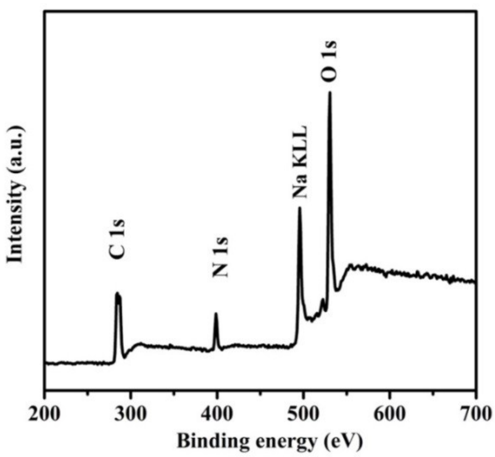

Fig. S2. The XPS survey scan of CNDs.

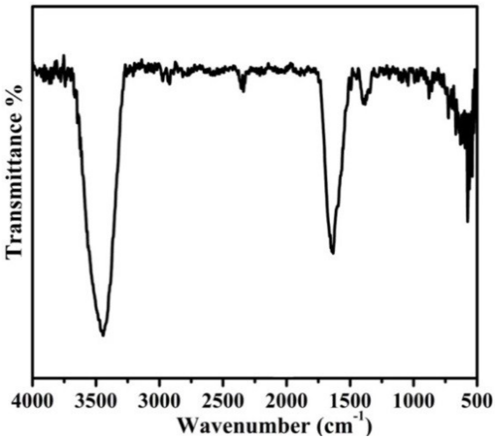

Fig. S3. FTIR spectra of CNDs.

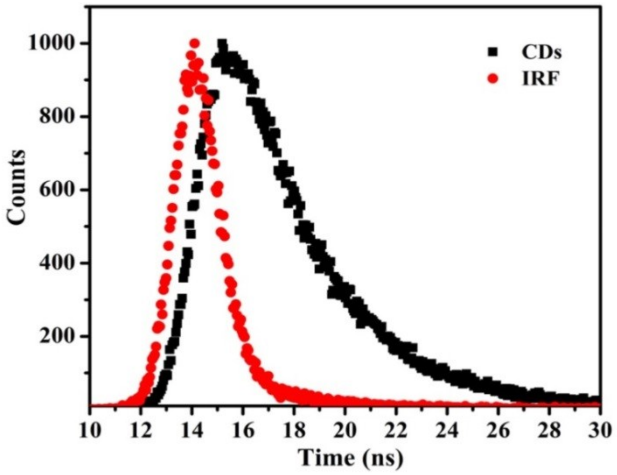

Fig. S4. Time resolved luminescence decay curves for CNDs (540 nm excitation) (IRF = instrument response function).

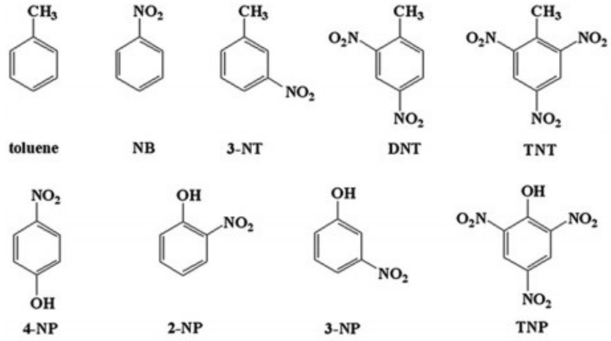

Fig. S5. The structure of nitroaromatics used in the present study

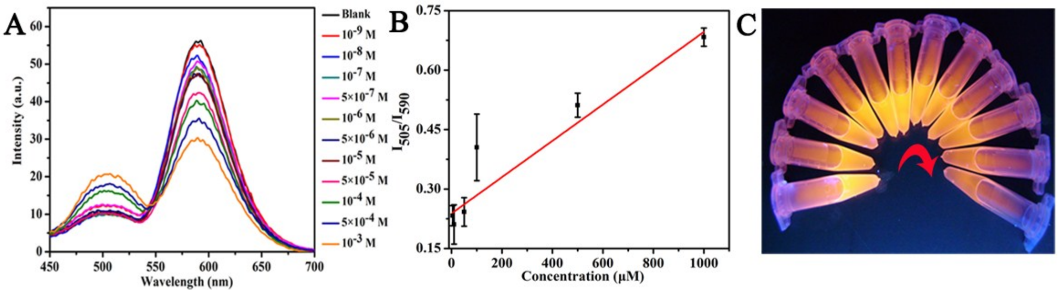

Fig. S6. (A) The evolution of fluorescence spectra of the mixture of CNDs with the addition of TNT. (B) Linear curve of TNT. (C) The photos showed the corresponding color evolutions under UV lamp.

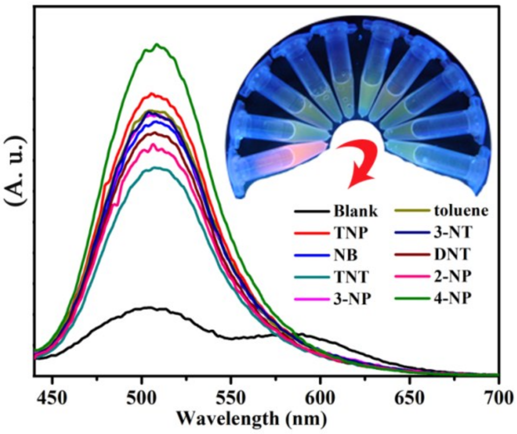

Fig. S7. Effect of nitroaromatic explosives on the fluorescence of CNDs (excitation wavelength 420 nm) under UV lamp for 20 min, the fluorescence spectra show the fluorescence that occurs upon addition of TNP to the solution containing CNDs and corresponding nitroaromatic explosives, respectively.

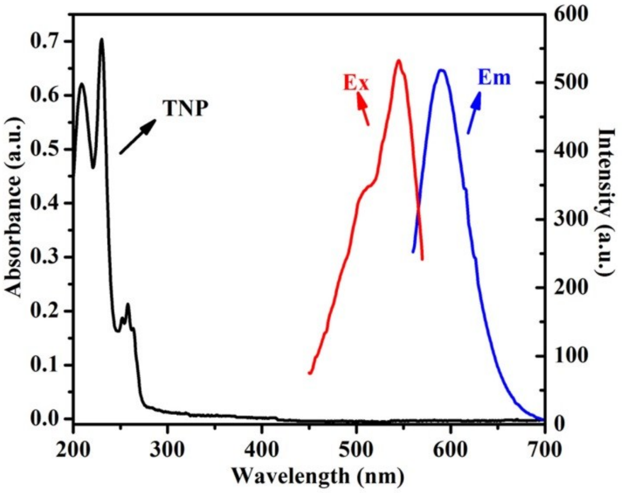

Fig. S8. Absorption spectra of TNP (black line), the excitation spectrum (red line) and the emission spectrum of CNDs (blue line).

Cell Culture: HeLa cells (human cancer cell lines) were grown in Dulbecco's Modified Eagle's

Medium (DMEM) with 10% fetal bovine serum (FBS), 1% penicillin and streptomycin. HeLa cells were seeded into 12-well plates and incubated for 24 h prior to treatment. Then CNDs and FA were added at a concentration equal to 0.03125 mg/ mL of pure dyes and incubated for 3 h at 37 °C with 5% CO_2. Then the cells were washed three with PBS and fixed with 2.5% glutaraldehyde for 10 min. Discard the glutaraldehyde solution and fully washed the cells with PBS three times. The cells were imaged after washing the culture medium three times with PBS. Then the cells observed under a Leica DM IL LED Fluorescence inverted microscope (FIM).

MTT Assay: HeLa cells were seeded into 96-well plates at a density of 1×10^4 per well in 100 μL of medium and grown overnight. Then the medium was replaced with medium contain 10, 20, 40, and 50 μg mL^{-1} CNDs, and incubated at 37 °C for 24 h. After that, 20 μL of 0.5 mg/mL MTT solution was added to each cell well. After incubation for 3 h, the medium was then replaced with dimethyl sulfoxide (DMSO: 150 μL) per well and the absorbance was monitored with a microplate reader (Bio-TekELx800) at a wavelength of 490 nm. The cytotoxicity was expressed as the percentage of cell viability relative to untreated control cells.

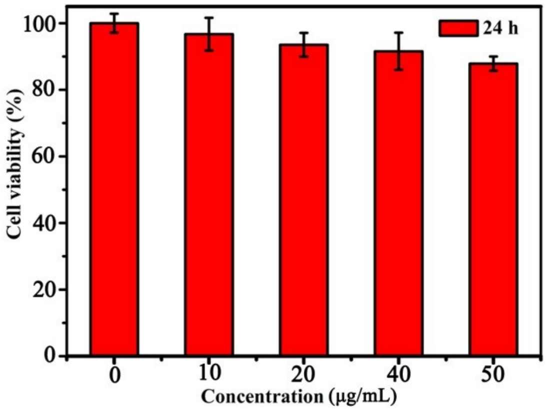

Fig. S9. Cell viability assays of HeLa cells treated with different concentrations of CNDs.
